# Rehabilitation in critically ill children: Findings from the Korean National Health Insurance database

**DOI:** 10.1371/journal.pone.0266360

**Published:** 2022-03-31

**Authors:** Joongbum Cho, Hyejeong Park, Danbee Kang, Esther Park, Chi Ryang Chung, Juhee Cho, Sapna R. Kudchadkar

**Affiliations:** 1 Department of Critical Care Medicine, Samsung Medical Center, Sungkyunkwan University School of Medicine, Seoul, Republic of Korea; 2 Center for Clinical Epidemiology, Samsung Medical Center, Seoul, Republic of Korea; 3 Department of Clinical Research Design & Evaluation, SAIHST, Sungkyunkwan University, Seoul, Republic of Korea; 4 Department of Anesthesiology and Critical Care Medicine, Charlotte R. Bloomberg Children’s Center, Johns Hopkins University School of Medicine, Baltimore, Maryland, United States of America; 5 Department of Pediatrics, Charlotte R. Bloomberg Children’s Center, Johns Hopkins University School of Medicine, Baltimore, Maryland, United States of America; 6 Department of Physical Medicine & Rehabilitation, Johns Hopkins University School of Medicine, Baltimore, Maryland, United States of America; University of Cape Town, SOUTH AFRICA

## Abstract

**Purpose:**

Intensive care unit (ICU) survivors suffer from physical weakness and challenges returning to daily life. With the importance of rehabilitating patients in the pediatric intensive care unit being increasingly recognized, we evaluated the prevalence of physical and occupational therapy (PT/OT)-provided rehabilitation and factors affecting its use.

**Methods:**

We conducted a retrospective cohort analysis of rehabilitation between 2013 and 2019 using the Korean National Health Insurance database. All patients aged 28 days to 18 years who had been admitted to 245 ICUs for more than 2 days were included. Neonatal ICUs were excluded.

**Results:**

Of 13,276 patients, 2,447 (18%) received PT/OT-provided rehabilitation during their hospitalization; prevalence was lowest for patients younger than 3 years (11%). Neurologic patients were most likely to receive rehabilitation (adjusted odds ratio [aOR], 6.47; 95% confidence interval [CI], 5.11–8.20). Longer ICU stay (versus ≤ 1 week) was associated with rehabilitation (aOR for 1–2 weeks, 3.50 [95% CI, 3.04–4.03]; 2–3 weeks, 6.60 [95% CI, 5.45–8.00]; >3 weeks, 13.69 [95% CI, 11.46–16.35]). Mechanical ventilation >2 days (aOR, 0.78; 95% CI, 0.67–0.91) and hemodialysis (aOR, 0.50; 95% CI, 0.41–0.52) were negatively affecting factors.

**Conclusion:**

Prevalence of rehabilitation for critically ill children was low and concentrated on patients with a prolonged ICU stay. The finding that mechanical ventilation, a risk factor for ICU-acquired weakness, was an obstacle to rehabilitation highlights the need for studies on early preventive rehabilitation based on individual patient needs.

## Introduction

Pediatric survivors of critical illness suffer from post-intensive care unit (ICU) syndrome, which encompasses physical, cognitive, and mental health impairments [1, 2]. Those impairments persist in 48% of survivors at 6 months and in 9% at 3 years after pediatric ICU (PICU) discharge [3, 4]. Impaired quality of life can make the return to daily life for children and families difficult [5, 6]. Physical impairments that are a consequence of critical illness and PICU stay can be caused by ICU-acquired weakness associated with sepsis, multiorgan dysfunction, and/or immobilization and common use of deep sedation [7, 8]. The effect of these impairments may be magnified in children who are actively growing and developing.

Early and progressive ICU rehabilitation is a promising therapy to prevent and mitigate the functional impairments that survivors of pediatric critical illness face [9]. Early physical therapy (PT)- and occupational therapy (OT)-provided mobilization increases return to independent functional status and decreases ICU-acquired weakness in adults [10, 11]. Recent studies in North America and Canada have shown that 33–35% of children who are admitted to the PICU for >72 hours receive PT/OT-provided mobilization on any given day, and that mobilization is overall feasible and safe [12–14].

Early mobilization is one of the key elements of the ABCDEF bundle, which improves ICU outcomes [15]. However, few studies carried out in Asian countries have focused on rehabilitation practices in critically ill children, practices that are essential for optimizing functional outcomes. We hypothesized that the prevalence of rehabilitation and mobilization is influenced by organizational structure, patient characteristics, and interventions.

Every year, 76 in every 100,000 Korean children are admitted to an ICU and are at risk of possible impairments [16]. Knowing that only 17% of adult ICU survivors receive any rehabilitation in Korea [17], we aimed to evaluate rehabilitation practices and factors associated with PT and/or OT involvement in children initially admitted to Korean PICUs.

## Methods

### Study population

We conducted a nationwide retrospective cohort analysis using the Health Insurance Review and Assessment (HIRA) database from January 1, 2013, to July 31, 2019. In Korea, all citizens are covered by the National Health Insurance Service or Medical Aid Program [18]. Claims submitted for reimbursement to the Korean National Health Insurance and Medical Aid Program are reviewed by the HIRA service, a central office in the Korean Ministry of Health. Therefore, the HIRA database used in this study covers all admissions except those with rare administrative errors or those for which HIRA rejected payment because the claim was deemed inappropriate.

**Fig 1:**
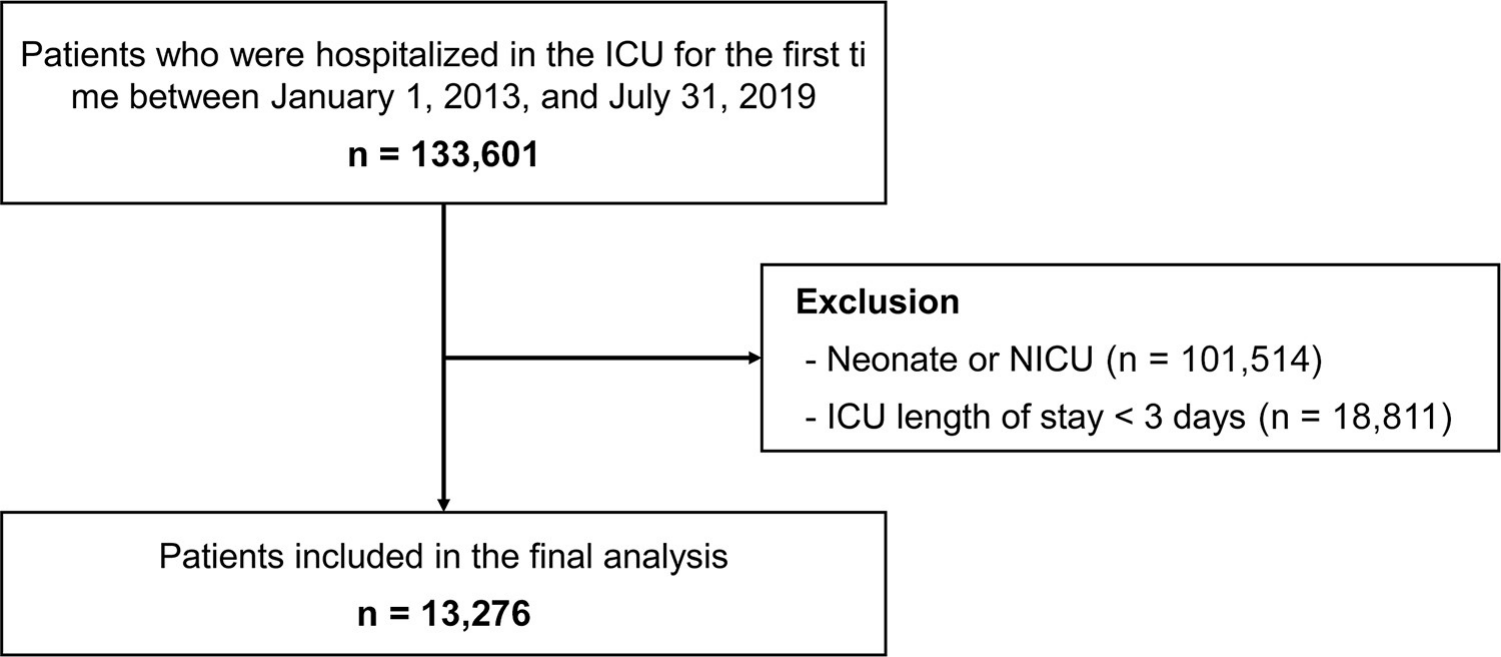
Study flow chart of patient inclusions and exclusions. ICU, intensive care unit; NICU, neonatal intensive care unit.

In our database study sample, we included patients younger than 18 years and admitted to the ICU at least once (N = 133,601). We defined ICU admission using the presence of mandatory claim codes (S1 Table) in the HIRA database. Consistent with previous studies, hospital stays separated by less than 2 days were considered the same hospital admission [19, 20]. We excluded neonatal (<28 days of age) admissions and patients who were admitted to neonatal ICUs because of substantial differences in neonatal rehabilitation care (N = 101,514). We also excluded patients who had an ICU stay shorter than 3 days (72 hours; N = 18,811) to focus on the patient group with the highest risk of physical impairments. In addition, 48 to 72 hours has been suggested as a threshold for early mobilization in previous studies [21]. In total, 13,276 patients were eligible for inclusion in the analysis (Fig 1). Each individual was matched with a permanent Korean resident registration number and identified without duplication. The matching identifiers were included in the HIRA claim data. The study was reviewed by the Institutional Review Board (IRB) of Samsung Medical Center (IRB protocol 2019-12-156), and informed consent was exempted because we accessed only previously collected de-identified administrative data.

### Measurements

The prevalence of each PT/OT-provided rehabilitation was evaluated by the presence of cost claim codes in the reimbursement requests during hospital admission (ICU or general ward). All types of rehabilitation claim codes were based on a specific therapist-provided session. PT was defined as the presence of PT claim codes. OT was defined as the presence of OT claim codes. We defined PT/OT-provided rehabilitation as the presence of either PT or OT codes (S1 Table).

Information on primary diagnosis, procedures, and prescriptions was based on HIRA claim codes. Primary diagnosis was defined as the condition primarily responsible for the patient’s need for treatment. This diagnosis was documented in accordance with the Korean Classification of Disease, 6^th^ edition, a modified version of the International Classification of Disease, 10^th^ edition [22]. In this classification, congenital heart diseases are classified into congenital anomalies, and cardiomyopathy/myocarditis are classified into circulatory diseases. Discharge outcomes were ICU length of stay (LOS) and in-hospital mortality.

Procedures of interest were evaluated according to Korean National Health Insurance procedure codes. Use of mechanical ventilation, high flow nasal cannula, extracorporeal membrane oxygenation, hemodialysis, a central venous catheter, or a Foley catheter was designated by claim codes (S1 Table). Because mechanical ventilation for a procedure or minor operation may affect patients’ mobilization differently, we divided the use of mechanical ventilation into two categories according to ventilation duration. We identified the use of vasopressor drugs, sedatives, and neuromuscular blockers using Korean Active Pharmaceutical Ingredients codes [23]. The vasopressors included dopamine, dobutamine, epinephrine, and norepinephrine; the sedatives included midazolam, fentanyl, remifentanil, ketamine, propofol, morphine, and hydromorphone; and the neuromuscular blockers were vecuronium, atracurium, rocuronium, and cis-atracurium.

Information on hospital characteristics was obtained from the HIRA Medical Care Institution database. Hospitals were categorized as tertiary hospitals, general hospitals, and other hospitals according to hospital bed and specialty numbers as defined by the Korean Health Law. Hospital region was categorized as capital (Seoul), metropolitan, or province. Metropolitan areas included Busan, Incheon, Daegu, Gwangju, Daejeon, and Ulsan. Bed-to-nurse grade was divided according to HIRA reimbursement classification and a previous study [20]. The bed-to-nurse grade was defined as the number of beds divided by the number of full-time equivalent registered nurses in a unit. As the bed-to-nurse grade increases, the number of patients cared for by a nurse increases.

### Statistical analysis

Patients were divided into two groups based on presence or absence of PT/OT-provided rehabilitation during hospitalization. We used mean and standard deviation or median and interquartile range to describe the distribution of continuous variables. Chi-squared tests and Student’s *t*-tests were used to compare categorical and continuous variables, respectively.

We conducted random-effects logistic regression to identify univariate and multivariable predictors for PT/OT-provided rehabilitation. Odds ratios (OR) with 95% confidence intervals (CI) were estimated using the model. We used univariate random-effects logistic regression analysis to identify possible predictors of PT/OT-provided rehabilitation. Then, we developed a multivariable model that included age, sex, tertiary hospital, ICU LOS, bed-to-nurse grade, emergency room, region, admission department, primary diagnosis, and procedures during hospital admission (mechanical ventilation, high flow nasal cannula, vasopressor drugs, sedative use, neuromuscular blocker use, placement of central venous catheter, Foley catheterization, extracorporeal membrane oxygenation, and hemodialysis).

We considered a p*-*Value < 0.05 as statistically significant for all analyses. Statistical analysis was carried out with SAS and Visual Analytics.

## Results

**Fig 2:**
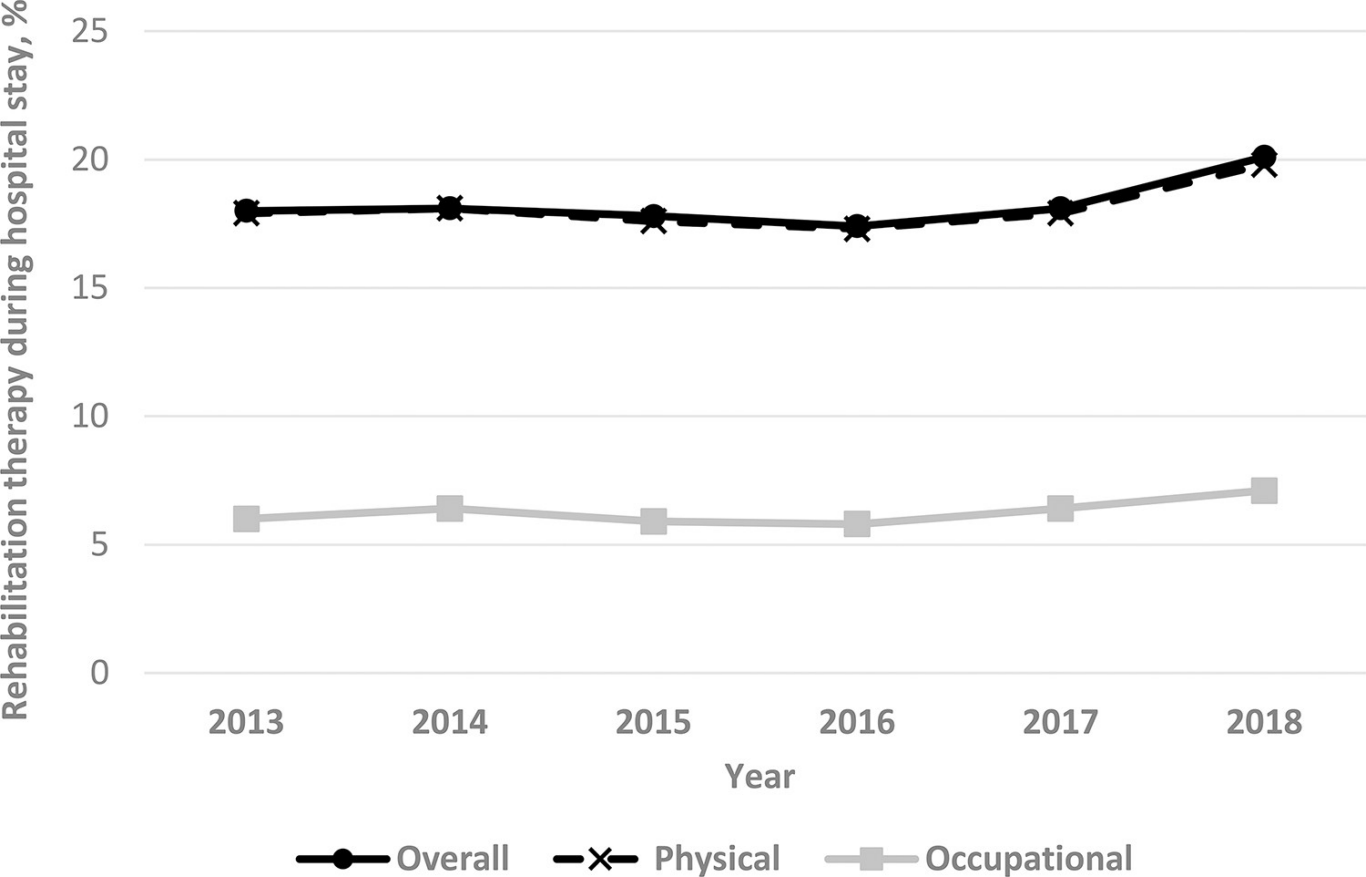
Trends in physical therapy/occupational therapy rehabilitation provided to critically ill children in Korean intensive care units over a 5-year period. ICU, intensive care unit. Overall p for trend < 0.01.

**Fig 3:**
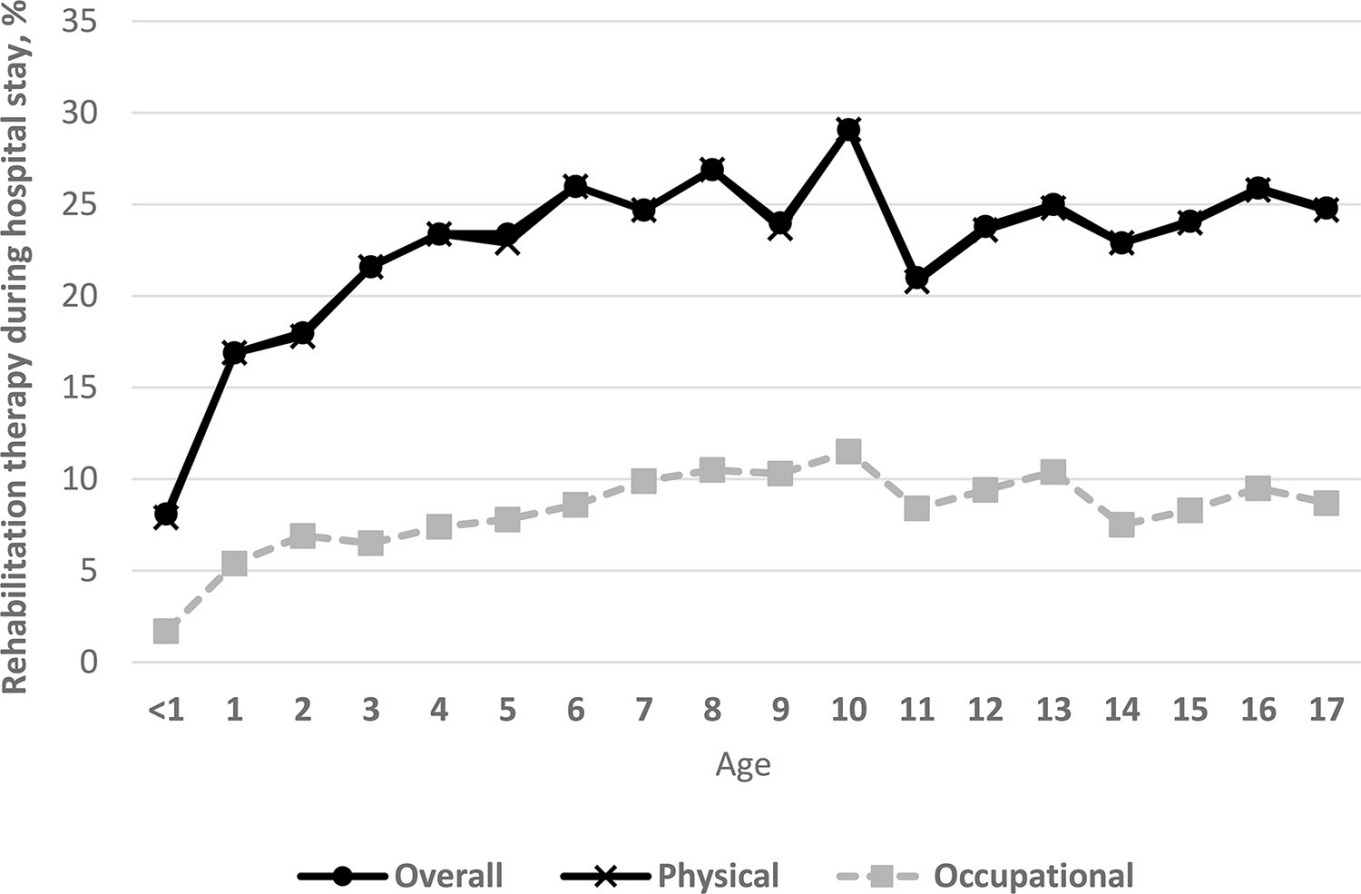
Distribution of physical/occupational therapy according to age in Korean intensive care units. ICU, intensive care unit. Overall p for trend < 0.01.

Of the 13,276 patients who met inclusion criteria and were admitted to 245 Korean ICUs, 2,447 (18%) received PT/OT-provided rehabilitation at any time during their hospital stay. PT was used more frequently (18% of pediatric patients in 2013 and 20% in 2018) than was OT (6% in 2013 and 7% in 2018). Provision of PT/OT increased significantly over time, from 18% to 20% between 2013 and 2018 (p for trend = 0.02; Fig 2). Additionally, PT/OT-provided rehabilitation increased with increasing patient age (p for trend < 0.01; Fig 3).

Univariate analyses of PT/OT-provided rehabilitation and demographic/admission characteristics were shown in Table 1. In an unadjusted comparison between patients who did and did not receive PT or OT, those who received PT/OT-provided rehabilitation were older (15 vs. 3 years, p < 0.01). Patients in the PT/OT-provided rehabilitation group were more likely to have been admitted to emergency rooms (73% vs. 60%, p < 0.01). There were significant proportional differences in the bed-to-nurse-grade ICUs (p < 0.01) and in the region of hospitals (p < 0.01). Primary diagnoses within the PT/OT-treated group differed from those in the group that did not receive rehabilitation (p < 0.01). Those in the PT/OT-treated group were more likely to have a neurologic disease (21% vs. 9%), injury (20% vs. 16%), or neoplasm (17% vs. 10%) (Table 1). In addition, patients that received PT/OT more frequently required procedures during hospital admission (p < 0.01). Median ICU LOS (14 vs. 5 days, p < 0.01) and hospital LOS (46 vs. 13 days, p < 0.01) were longer for those who received PT/OT-provided rehabilitation than for those who did not (Table 2).

**Table 1:** Characteristics of ICU patients according to PT/OT-provided rehabilitation in Korea, January 2013 to July 2019.

Variables	Overall (N = 13,276)	PT/OT-provided rehabilitation (N = 2,447)	No PT/OT-provided rehabilitation (N = 10,829)	p-Value
Age (years), median (IQR)	5 (1–13)	15 (3–15)	3 (1–13)	<0.01
Sex, male	7,698 (58)	1,450 (59)	6,248 (58)	0.16
Tertiary hospital	9,748 (73)	1,833 (75)	7,915 (73)	0.18
Bed-to-nurse grade				<0.01
1	6,405 (48)	1,238 (50)	5,167 (48)	
2	4,220 (31)	791 (32)	3,429 (32)	
3	1,748 (13)	280 (11)	1,468 (14)	
4 or above	903 (7)	138 (6)	765 (7)	
Emergency room	8,296 (62)	1,788 (73)	6,508 (60)	<0.01
Region				<0.01
Capital	5,262 (40)	1,129 (46)	4,133 (38)	
Metropolitan	3,285 (25)	537 (22)	2,748 (25)	
Province	4,729 (36)	781 (32)	3,948 (37)	
Admission department^a^				0.20
Surgical	5,101 (38)	968 (40)	4,133 (38)	
Medical	8,175 (62)	1,479 (60)	6,696 (62)	
Primary diagnosis				<0.01
Congenital anomaly	2,892 (22)	181 (7)	2,711 (25)	
Injury	2,230 (17)	485 (20)	1,745 (16)	
Neoplasm	1,503 (11)	413 (17)	1,090 (10)	
Respiratory	1,471 (11)	135 (6)	1,336 (12)	
Neurologic disease	1,539 (12)	522 (21)	1,017 (9)	
Circulatory disease	1,328 (10)	428 (18)	900 (8)	
Gastrointestinal disease	386 (3)	32 (1)	354 (3)	
Not otherwise classified	393 (3)	32 (1)	361 (3)	
Infectious disease	444 (3)	53 (2)	391 (4)	
Other	1,090 (8)	166 (7)	924 (9)	

All values except for age are presented as frequency (%).

ICU, intensive care unit; PT, physical therapy; OT, occupational therapy; IQR, interquartile range.

^a^Medical includes general medicine, internal medicine, neurology, psychiatry, pediatrics, dermatology, radiology, radiation oncology, clinical pathology, tuberculosis, rehabilitation medicine, family medicine, emergency medicine, industrial medicine, preventive medicine, and conservative dentistry. Surgical includes general surgery, orthopedic surgery, neurosurgery, thoracic and cardiovascular surgery, plastic surgery, anesthesiology, obstetrics and gynecology, ophthalmology, otorhinolaryngology, urology, and oral surgery.

**Table 2:** Required procedures and hospital outcomes of ICU patients according to PT/OT-provided rehabilitation in Korea, January 2013 to July 2019.

Procedures and hospital outcomes	Overall (N = 13,276)	No PT/OT-provided rehabilitation (N = 10,829)	PT/OT-provided rehabilitation (N = 2,447)	p-Value
Required procedures				
Mechanical ventilation	6.970 (53)	5,365 (50)	1,606 (66)	<0.01
1–2 days	2,208 (17)	1,959 (18)	249 (10)	
>2 days	4,762 (36)	3,405 (31)	1,357 (56)	
HFNC	1,595 (12)	1,221 (11)	374 (15)	<0.01
Vasopressors	7,988 (60)	6,213 (57)	1,775 (73)	<0.01
Sedatives	9,793 (74)	7,605 (70)	2,188 (89)	<0.01
Neuromuscular blocker	5,610 (42)	4,310 (40)	1,300 (53)	<0.01
Central venous catheterization	3,824 (29)	2,695 (25)	1,129 (46)	<0.01
Foley catheterization	5,114 (39)	4,070 (38)	1,044 (43)	<0.01
ECMO	333 (3)	182 (2)	151 (6)	<0.01
Hemodialysis	864 (7)	641 (6)	223 (9)	<0.01
Hospital outcomes				
ICU length of stay, median (IQR)	5 (3–10)	5 (3–8)	14 (7–30)	<0.01
≤1 week	8708 (66)	8010 (74)	698 (29)	
1–2 weeks	2274 (17)	1689 (16)	585 (24)	
2–3 weeks	811 (6)	485 (5)	326 (13)	
>3 weeks	1484 (11)	645 (6)	839 (34)	
Hospital LOS, days, median (IQR)	15 (9–29)	13 (8–21)	46 (26–84)	<0.01
Hospital mortality (%)	1,009 (8)	808 (8)	201 (8)	0.21

All values are presented as frequency (%) except where indicated.

ICU, intensive care unit; PT, physical therapy; OT, occupational therapy; HFNC, high flow nasal cannula; ECMO, extracorporeal membrane oxygenation; IQR, interquartile range; LOS, length of stay.

In a multivariable model (Table 3), older patients (adjusted OR [aOR] for every 1 year, 1.05; 95% CI, 1.04–1.06) were more likely to receive PT/OT-provided rehabilitation during their hospital stay, as were patients admitted from emergency rooms (aOR, 1.27; 95% CI, 1.11–1.45) and medical departments (aOR, 1.24; 95% CI, 1.08–1.42). Patients with an ICU LOS longer than 1 week were more likely than those with an LOS < 1 week to receive PT/OT (aOR for 1–2 weeks, 3.50; 95% CI, 3.04–4.03; aOR for 2–3 weeks, 6.60; 95% CI, 5.45–8.00; and aOR for > 3 weeks, 13.69; 95% CI, 11.46–16.35). Patients admitted for neurologic disease (aOR, 6.47; 95% CI, 5.11–8.20), circulatory disease (aOR, 5.29; 95% CI, 4.18–6.70), or injury (aOR, 3.94; 95% CI, 3.80–5.05) were more likely to receive PT/OT than were patients with a congenital anomaly.

**Table 3:** Odds ratio (95% CI) for PT/OT-provided rehabilitation of critically ill children in intensive care units in Korea, 2013 to 2019.

Predictors of PT/OT-provided rehabilitation	Crude odds ratio (95% CI)	p-Value	Adjusted odds ratio (95% CI)^a^	p-Value
Age, years	1.07 (1.06–1.07)	<0.01	1.05 (1.04–1.06)	<0.01
Sex, male	1.07 (0.98–1.17)	0.16	1.00 (0.90–1.25)	0.94
ICU length of stay				
≤1 week	Reference		Reference	
1–2 weeks	3.98 (3.52–4.49)	<0.01	3.50 (3.04–4.03)	<0.01
2–3 weeks	7.71 (6.57–9.05)	<0.01	6.60 (5.45–8.00)	<0.01
>3 weeks	14.91 (13.11–16.96)	<0.01	13.69 (11.46–16.35)	<0.01
Tertiary hospital (ref: others)	1.10 (0.99–1.22)	0.07	1.04 (0.80–1.48)	0.70
Bed-to-nurse grade				
1	Reference		Reference	
2	0.96 (0.87–1.06)	0.45	0.96 (0.80–1.16)	0.69
3	0.80 (0.69–0.92)	<0.01	1.05 (0.82–1.33)	0.72
4 or above	0.75 (0.62–0.91)	<0.01	0.92 (0.67–1.24)	0.57
Emergency room	1.80 (1.63–1.99)	<0.001	1.27 (1.11–1.45)	<0.01
Region				
Capital	Reference		Reference	
Metropolitan	0.72 (0.64–0.80)	<0.01	0.70 (0.54–0.89)	<0.01
Province	0.72 (0.66–0.80)	<0.01	0.70 (0.56–0.89)	<0.01
Admission department, medical	1.06 (0.97–1.16)	0.20	1.24 (1.08–1.42)	<0.01
Primary diagnosis				
Congenital anomaly	Reference		Reference	
Injury	4.16 (3.47–4.99)	<0.01	3.94 (3.08–5.05)	<0.01
Neoplasm	5.68 (4.70–6.85)	<0.01	3.60 (2.87–4.52)	<0.01
Respiratory	1.51 (1.20–1.91)	<0.01	1.70 (1.28–2.27)	<0.01
Neurologic disease	7.69 (6.40–9.24)	<0.01	6.47 (5.11–8.20)	<0.01
Circulatory disease	7.12 (5.89–8.61)	<0.01	5.29 (4.18–6.70)	<0.01
Gastrointestinal disease	1.35 (0.92–2.00)	0.13	1.15 (0.75–1.78)	0.52
Not elsewhere classified	1.33 (0.90–1.96)	0.16	2.04 (1.29–3.20)	0.01
Infectious disease	2.03 (1.47–2.81)	<0.01	2.55 (1.73–3.75)	<0.01
Other	2.69 (2.15–3.37)	<0.01	2.84 (2.17–3.71)	<0.01
Procedures during hospital admission				
Mechanical ventilation				
No	Reference		Reference	
1–2 days	0.83 (0.71–0.96)	0.01	0.89 (0.75–1.07)	0.20
>2 days	2.59 (2.35–2.85)	<0.01	0.78 (0.67–0.91)	<0.01
HFNC	2.07 (1.77–2.42)	<0.01	1.27 (1.08–1.50)	<0.01
Vasopressor drugs	1.71 (1.55–1.89)	<0.01	0.94 (0.82–1.08)	0.41
Sedative use	3.48 (3.16–3.83)	<0.01	2.51 (2.13–2.96)	<0.01
Neuromuscular blocker use	2.78 (2.35–3.29)	<0.01	1.29 (1.14–1.47)	<0.01
Central venous catheterization	2.59 (2.36–2.83)	<0.01	1.30 (1.15–1.47)	<0.01
Foley catheterization	1.24 (1.13–1.35)	<0.01	1.38 (1.23–1.56)	<0.01
ECMO	4.35 (3.38–5.60)	<0.01	1.39 (1.05–1.83)	0.02
Hemodialysis	2.03 (1.70–2.42)	<0.01	0.50 (0.41–0.62)	<0.01

PT, physical therapy; OT, occupational therapy; CI, confidence interval; ICU, intensive care unit; HFNC, high flow nasal cannula; ECMO, extracorporeal membrane oxygenation.

Adjusted for age, sex, tertiary hospital, ICU length of stay, bed-to-nurse grade, emergency room, region, admission department, primary diagnosis, procedures during hospital admission (mechanical ventilation, HFNC, vasopressor drugs, sedative use, neuromuscular blocker use, placement of central venous catheter, Foley catheterization, ECMO, hemodialysis).

^a^Random-effects logistic regression model.

Patients who used mechanical ventilation for more than 2 days (aOR: 0.78; 95% CI: 0.67–0.91) and hemodialysis (aOR: 0.50, 95% CI: 0.41–0.62) were less likely to receive PT/OT-provided rehabilitation.

## Discussion

In this national Korean cohort study, the prevalence of inpatient PT/OT-provided rehabilitation for critically ill children was low and varied widely according to ICU LOS. In addition, patient demographics, procedures, and hospital characteristics were associated with PT/OT-provided inpatient rehabilitation. Our investigation into current implementation of pediatric critical care rehabilitation, a subject rarely studied in Asian countries, suggests that current practice status may not be enough in amount and may not meet clinical timeliness.

We found that critically ill children with a longer ICU LOS had more frequent PT/OT-provided rehabilitation. Longer ICU LOS might have been the reason for the higher prevalence of PT/OT involvement, as muscle strength decreases with increased duration of bed rest [24]. An increase in PICU LOS also is associated with higher severity scores and decreased performance scores [25]. However, the effect of LOS on prevalence could relate to insufficient PT/OT-provided rehabilitation resources. In Korea, rehabilitation resources are insufficient for the number of pediatric patients [26]. The shortage may be reflected in this study’s low prevalence of PT/OT (18%). Therefore, timely rehabilitation in the acute phase is not ensured. Nevertheless, early rehabilitation in the acute phase of critical illness is important for decreasing ICU LOS and improving muscle strength and functional status [11, 27, 28]. Our therapy goal should be to prevent complications, not to implement rehabilitation for patients only after complications have been established. A central question is whether the ease of rehabilitation or requirement of rehabilitation is the main factor in its initiation. Data on the first day of rehabilitation were unavailable for this study. Additional studies into the effect of ICU LOS on early ICU rehabilitation are required.

Patient age and diagnosis affected the prevalence of PT/OT-provided rehabilitation. Younger children received less frequent PT/OT, as reported in other studies [12–14]. However, younger children do not necessarily require less rehabilitation than older children, particularly because they are undergoing rapid neurologic and physical development. Pereira et al. [29] reported that 82% of young children (median age 19 months) exhibited an alteration in functional status score after ICU discharge and that their median overall score was categorized as moderate dysfunction. In addition, infants’ and toddlers’ rapid development should be assessed closely throughout an inpatient stay. Such children might require PT/OT to recover from developmental delay [30]. Low rehabilitation application at a young age may be due to the complexity of ongoing developmental changes and difficulties in early detection [31]. It may come from barriers of therapists such as limited specialized training, lack of collaboration, or policy limitations [32].

Diagnosis of congenital anomaly was a negative predictor of PT/OT-provided rehabilitation. Most patients with a congenital anomaly were infants or younger children [19]. Therefore, their low rate of OT/PT-provided rehabilitation might be associated with their young age. Congenital heart disease was the most common congenital anomaly [19]. However, children undergoing congenital heart surgery are vulnerable to impaired functional status [33]. Neurologic abnormality was the most common primary diagnosis for those who received PT/OT in this study. Neurologic patients have low baseline functional status [34]. Therefore, their low functional status may be easily screened, and their frequent rehabilitation may be appropriate practice.

Various procedures can affect inpatient rehabilitation implementation. Use of mechanical ventilation for more than 2 days and renal replacement therapy were positive predictors in unadjusted analysis. However, both procedures turned into negative predictors of PT/OT-provided rehabilitation when we adjusted for the length of ICU stay in addition to other conditions. It is possible that patients under one of these 2 procedures were more severely ill, so they stayed longer and received more rehabilitation than the other patients. However, when the length of ICU stays was equal, these patients received less rehabilitation compared to the patients without these 2 procedures. Endotracheal intubation and other invasive devices were reported as a barrier to out-of-bed mobility in previous studies, but the effect on overall in- and out-of-bed PT/OT was variable [12–14]. The lack of rehabilitation services for these patients might be associated with concerns over dislodgement of devices, which could cause life-threatening adverse events.

However, ventilated patients have a greater need for PT/OT-provided rehabilitation than do other patients. Long-term mechanical ventilation is associated with higher functional impairments and more critical illness-related neuromuscular abnormalities [7, 29]. Muscle loss and functional impairments persist for more than 1 year in patients who have experienced respiratory failure [6]. According to expert consensus, mechanically ventilated adults are safe to be mobilized if they have a fraction of inspired oxygen less than 0.6 with blood oxygen saturation greater than 90% and a respiratory rate less than 30 breaths/minute [35]. Guidance for mechanically ventilated children is required before PT/OT requirements can be met for pediatric patients in the ICU. Other procedures such as high flow nasal cannula usage, central venous catheter placement, and vasopressor usage were predictive factors of PT/OT in this study (Table 3). We suspect that the requirement for these procedures indicates higher disease severity that results in a higher rehabilitation requirement.

Hospital organization can affect rehabilitation prevalence, and nursing has a central role in rehabilitation [36]. In previous studies, nurses performed 37% to 48% of mobilization in the absence of rehabilitation therapists in PICUs [12–14]. In this study, the aOR of PT/OT-provided rehabilitation was not statistically different based on bed-to-nurse grades. We could not ascertain the prevalence of nurse-only mobilization. Participation of nurses in PT/OT-provided rehabilitation can differ according to ICU culture and affects the prevalence of rehabilitation.

Our study has several limitations. First, because the database used in this study was from claims for insurance reimbursements, we could not evaluate important parameters such as patient severity or level of PT/OT-provided rehabilitation. In addition, no information was provided regarding the initial hospital day or location of PT/OT-provided rehabilitation. It was difficult to distinguish whether PT/OT-provided rehabilitation was a preventive measure or initiated after observation of functional impairments. Therefore, additional studies will be needed to determine the prevalence and predictors of early rehabilitation in critically ill children. Second, this study was conducted in a country that has a national health insurance system. Therefore, the results might not be generalizable to other countries with different rehabilitation costs or resource settings. However, we evaluated all children admitted to the ICU, which is crucial for a prevalence study. In addition, our findings suggested predictive factors for PT/OT-provided rehabilitation and a possible discrepancy between feasibility and requirements of PT/OT-provided rehabilitation in critically ill children.

In conclusion, prevalence of rehabilitation for critically ill children is low in Korean ICUs and affected by ICU duration and device support, raising concerns about whether the clinical needs of patients are being met. Additional studies on the timely and systematic implementation of implementation of therapies to meet rehabilitation needs of these critically ill patients are urgently needed.

## Supporting information

S1 Table Claim codes for treatments or procedures. (DOCX)

## Abbreviations

HIRA: Health Insurance Review and Assessment
ICU: intensive care unit
LOS: length of stay
OT: occupational therapy
PICU: pediatric intensive care unit
PT: physical therapy
