# Supplementary material for: Rehabilitation in critically ill children: Findings from the Korean National Health Insurance database
Source: PLoS One. 2022 Mar 31;17(3):e0266360. doi: 10.1371/journal.pone.0266360 (PMC8970491; doi:10.1371/journal.pone.0266360)
Supplement: S1 Table — (DOCX) [file pone.0266360.s001.docx]

**S1 Table. Claim codes for treatments or procedures**

| **Treatment/procedure** | **Health Insurance Review and Assessment claim codes** |
| --- | --- |
| Intensive care unit admission | AJ001-AJ590900 |
| Physical therapy | MM101, MM102, MM105, MM301, MM302 |
| Occupational therapy | MM111, NM112, NM113, MM114 |
| Mechanical ventilation | M5850, M5857, M5858, M5860 |
| High flow nasal cannula | M0046 |
| Extracorporeal membrane oxygenation | O1901, O1902, O1903, O1904 |
| Hemodialysis | O7020, O7031-O7034, O7051-O7054, O7062 |
| Central venous catheter | O1650, O1651, O1654-O1659, O2651-O2653, OA654 |
| Foley catheter | M0060, M0065 |
